# Development and short evaluation of the Dutch healthy diet index for pregnant women; DHD-P

**DOI:** 10.3389/fnut.2024.1386888

**Published:** 2024-04-26

**Authors:** Janine P. M. Faessen, Edith J. M. Feskens, Elske M. Brouwer-Brolsma

**Affiliations:** Division of Human Nutrition and Health, Wageningen University & Research, Wageningen, Netherlands

**Keywords:** diet quality, pregnancy, evaluation, maternal diet, diet index

## Abstract

**Introduction:**

Diet quality indices provide a quick indicator of overall diet and are commonly used in research and surveillance. We developed a Dutch Healthy Diet for pregnant women (DHD-P) index, comprising 22 components aligned with the 2021 Dutch food-based dietary guidelines for pregnant women. Our evaluation focused on assessing its performance and sensitivity to change.

**Methods:**

The DHD-P index was quantified by using a validated Food Frequency Questionnaire (FFQ) and two 24-h recalls at 12 and 24 weeks gestation completed by 24-to-41 year old pregnant women participating in the GLIMP-II study. Strength and direction of associations were evaluated based on de-attenuated correlation coefficients between FFQ and 24-h recall data at 24 weeks gestation (*n* = 47). Sensitivity to change was evaluated by comparing DHD-P index data assessed by both FFQ and recalls at 12 and 24 weeks gestation using paired t-tests or Wilcoxon Signed Rank Test (*n* = 27).

**Results:**

De-attenuated correlation coefficients between FFQ and 24-recall data showed a good correlation for the total DHD-P score (rho = 0.57) and moderate to good correlations for component scores. FFQ as well as recall data showed comparable dietary intake at 12 and 24 weeks, suggesting minimal changes during pregnancy. Correlations over time were moderate-to-good for scores based on FFQ and low to moderate for scores based on 24hRs, indicating better reproducibility of scores based on FFQ data.

**Conclusion:**

Considering the moderate to good correlations, the DHD-P index appears to be an appropriate index to assess diet quality among pregnant women, and could serve as a foundation to provide dietary feedback toward healthier food choices. Studies including dietary data for all relevant food groups and nutrients are needed to substantiate our findings and further explore the DHD-P sensitivity to change.

## Introduction

A diet quality index is a convenient and effective indicator of overall diet quality (1, 2). These indices capture information about a group of dietary components and are assumed to partially account for synergistic interactions between nutrients and/or food groups (3). Consequently, epidemiological studies are placing growing emphasis on exploring associations between diet quality indices and specific health outcomes, moving beyond a focus solely on single nutrients and foods. To illustrate, a meta-analysis on 18 studies concluded that a one unit higher maternal diet quality was associated with a one unit better child neurodevelopment score (1). Moreover, a low Healthy Eating Index 2015 score (<70) among 762 US pregnant women was associated with a higher risk of fetal growth restrictions and hypertensive disorders during pregnancy (RR = 0.33, 95%CI: 0.13–0.68 and RR = 0.46, 95%CI: 0.24–0.79) (4). In a study among 660 pregnant Mexican women, women in the highest tertile of the Maternal Diet Quality Score compared to lowest tertile had a reduced risk of a baby with low birth weight (OR = 0.22, 95%CI: 0.06–0.75) (2).

In addition to its application in epidemiological contexts, a diet quality index is a straightforward parameter to understand and apply by health care professionals, policy makers as well as the general population (3). Therefore, diet quality indices are valuable tools as part of mobile Health (mHealth)-applications to facilitate self-monitoring and providing more individualized feedback in clinical practices to improve diet quality (3, 5). In fact, diet quality indices improved dietary habits of users in 60% of previously reviewed apps (6). However, mHealth-applications using diet quality indices for pregnant women are currently not available.

A diet quality index can consist of several different components (e.g., food groups, macro or micronutrients), scoring methods (e.g., ratios, moderation, and optimum), weighting of components (e.g., dependent on health effects) and can be based on different dietary recommendations (5, 7). While several diet quality indices have been developed for pregnant women across the globe (8–11), no diet quality exists yet for Dutch pregnant women. However, there is a Dutch Healthy Diet 2015 (DHD-2015) index based on the Dutch dietary guidelines of 2015 for the general adult population, excluding pregnant and lactating women (5, 12). More recently (2021), the Health Council of the Netherlands developed dietary guidelines for pregnant women (13).

The main objective of this study was to refine the existing DHD-15 index, aligning it with the latest Dutch dietary recommendations for pregnant women (DHD-P), using data of the GLIMP-II study. This refinement serves as a crucial step in establishing the foundation for a personalized nutrition app tailored to the specific dietary needs of pregnant women. Subsequently, we studied the correlation between the DHD-P index calculated using food frequency questionnaire (FFQ) data and using 24-h recall (24hR) data, and examined the index’s ability to detect changes over time at 12 and 24 weeks of gestation.

## Methods

### Study design

Data were extracted from the GLIMP-II study (14, 15), a prospective cohort study to assess the impact of diet, nutrient status and other lifestyle factors on the development of gestational diabetes mellitus. Women visited the hospital at four timepoints: before pregnancy (T0), at 12 weeks of gestation (T1), at 24 weeks of gestation (T2) and after pregnancy (T3). At each time point, information on anthropometrics was obtained, followed by fasting venipuncture and a 75-grams oral glucose tolerance test including a venipuncture 2 h after glucose load. Furthermore, participants completed a FFQ and various questionnaires addressing lifestyle, health, and pregnancy-related factors at each time point. Additionally, participants submitted two 24hRs during each time period. The Medical Ethics Committee of Wageningen University & Research approved the GLIMP2 study (NL50554.081.14). All women gave their written informed consent before the start of the study.

### Study population

Between June 2015 and May 2017, women were enrolled in this study if they expressed a desire to become pregnant within 1 year or were less than 24 weeks pregnant. Recruitment took place through three hospitals in the eastern part of the Netherlands: Gelderse Vallei Hospital (Ede), Rijnstate (Arnhem), and Slingeland (Doetinchem). Inclusion criteria involved women aged 18–40, proficient in speaking and reading Dutch, and capable of independent decision making. Participants meeting any of the following criteria were excluded from the study: inability to read or speak Dutch, multiple gestation index pregnancy (e.g., twins), diagnosis of Type 1 diabetes mellitus, diagnosis of Type 2 diabetes mellitus, index pregnancy resulting in preterm birth (<32 weeks), and index pregnancy resulting in low birth weight (<2,500 g). Eventually, 107 women participated in the study, but not all provided complete dietary data at all timepoints, leading to varied inclusions for the different analyses performed.

### Dietary assessment

Dietary intake data were collected by using FFQ and 24hR. The semi-quantitative FFQ consisting of 173 items was validated to estimate habitual intake of energy, macronutrients, fiber, and B-vitamins among Dutch adults (16, 17). Consumption frequencies and number of units eaten or portion sizes were defined according to Dutch household measures (18). Frequencies ranged from ‘not in this month’ to ‘6–7 days per week’. Daily intake levels were calculated by multiplying frequency, portion size and nutrient content per gram using Dutch food composition table from 2011 (19). Additionally, two web-based 24hRs using Compl-eat™ (20) were completed for each time period. The tool guides participants in reporting all foods and drinks consumed during the previous day, including foods and standard recipes commonly consumed by the Dutch population. Portion sizes were reported in household measures, standard portions, weight in grams and volume in liters. Lastly, it reminds participants to report commonly forgotten foods such as snacks, sugar in coffee and cooking fats. Dates were randomly selected. Trained dieticians checked all reported 24hRs on unusual portion sizes and data completeness. Nutrient intakes were calculated using the Dutch Food Composition Database (NEVO) 2016. Participants reported their supplement usage at each time point, detailing frequency, quantity (number of tablets or drops), type, and brand. Supplement content was determined from product label information or the Anatomical Therapeutic Chemical (ATC) code website. For FFQ and recall comparison, women with data from both FFQ and two recalls at 24 weeks pregnancy were considered (*n* = 47). For the assessment of sensitivity to change, data of 27 women with FFQ and two 24hRs at both 12 and 24 weeks of pregnancy could be used.

### Development of the DHD-P index

Scoring of the DHD-P index was based on dietary recommendations for pregnant women and the general Dutch population (13, 21). The recommended minimum, maximum or range of intake from the guidelines informed the scoring for each component. If a recommended level or range was lacking, the score was assigned based on the perceived positive or negative health associations of the food/nutrient, drawing from existing literature and considering the population’s intake range. In the subsequent sections, we elaborate on the calculation for each modified or added component in the DHD-P index (i.e., iron, dairy, fish, caffeine, alcohol, soy products folic acid supplementation, vitamin D supplementation, vitamin A, iodine and iron) as compared to the original DHD index. As the scoring methodology for fruit, vegetables, whole grains, legumes, nuts, fats and oils, red meat, processed meat, coffee, sweetened beverages and fruit juices and salt is similar to the original DHD index (5), the calculation for these components is not further detailed in this manuscript except for Table 1.

**Table 1:** Overview of scoring components of the DHD-P index.

	Component	Dietary recommendation	Included	Excluded	Minimum score	Maximum score
1	Vegetables	At least 200 g per day	Vegetables (fresh, frozen, canned)		No intake	200 g or more
2	Fruit	At least 200 g per day	Fruits (fresh, frozen, canned)	Dried fruit, fruit juices	No intake	200 g or more
3	Whole grain products	At least 90 g of whole grain and replace refined cereals with wholegrain products	Bread, crackers, cereals, rice, pasta, noodles, containing 25% wholegrain flour	No snacks made from cereals/flour	No intake whole grains. Ratio 15th and 85th percentile	90 g or more whole grains. Ratio 15th and 85th percentile
4	Legumes	One portion per week	legumes		No intake	10 g per day or more
5	Nuts	At least 15 g per day (unsalted)	Nuts, seeds unmodified/modified		No intake	15 g a day or more
6	Dairy	Consume about 3 to 4 portions per day (450–600 g). Maximum of 40 g of cheese	Milk, milk products, plant-based protein drinks and desserts, fermented milk products, cheese, cheese replacements		No intake or > 750 g per day	450–600 g per day
7	Fish	Twice a week fish, of which one time a week fatty fish and one time a week lean fish.	Fish (lean, fat), crustaceans, shellfish		No consumption	At least 30 g fish per day or more and at least 15 g fatty fish per day
8	Fats and oils	Replace butter, hard margarines and cooking fats by soft margarines, liquid cooking fats and vegetable oils	Butter, soft/hard margarines, soft/hard cooking fats, vegetable oils		No consumption of soft margarines, liquid cooking fats and vegetable oils OR ratio of liquid cooking fats to solid cooking fats = <0.6	No consumption of butter, hard margarines and cooking fats OR. Ratio of liquid cooking fats to solid cooking fats = > 13
9	Red meat	Maximum 300 g per week	Unprocessed red meat	Raw meat, Processed meat	45 g per day or more	No intake
10	Processed meat	No consumption, maximum of 50 g per day	Processed meat	Raw meat	50 g per day or more	No intake
11	Sweetened beverages/fruit juices	Minimize consumption	Fruit/vegetable juices. Sweetened beverages	Alcoholic beverages	1 glass or more (250 g) per day	Less than 1 glass per day
12	Alcoholic beverages	No consumption	Alcoholic beverages	Beverages with <0.5% alcohol	Any amount of consumption	No consumption
13*	Coffee	Replace unfiltered coffee by filtered coffee	Filtered coffee	Unfiltered coffee	Any consumption of unfiltered coffee	Consumption of only filtered coffee or no coffee consumption
14	Caffeine	No more than 200 mg of caffeine (calculated from coffee and tea consumption)	Black or green tea. Filtered coffee (potentially including milk or sugar)	Other herbal/fruit teas, unfiltered coffee	More than 200 mg of caffeine per day	Less than 200 mg caffeine and only filtered coffee
15*	Salt	Limit consumption to 6 g/day (2.4 g/day of sodium)	Salt in products	Salt added during cooking or at the dinner table	> = 3.8 g/day Na	<=1.9 g/day Na
16	Soy products	Limit soy consumption to no more than 4 portions of soy milk/yoghurt per day and no more than 2 portions of soy products per week. Unless you do not consume soy milk/yoghurt, then there is no limitation on soy products.	Soy products (tempeh, tofu, etc.) and soy drinks		More than 4 portions per day of soy milk/yoghurt and/or more than 2 portions per week of soy products (if any soy milk/yoghurt is consumed)	Consumption below 4 portions per week of soy milk/yoghurt and below 2 portions of soy products per week (if any soy milk/yoghurt is consumed)
17	Unhealthy foods	Limit consumption of unhealthy day and week choices	Sweet spreads, cakes, cookies, chips or pretzels, chocolate, savory snacks, sauces, and use of sugar in coffee or tea.	Products that were already included in one of the other components (e.g., orange juice under component 15)	>7 week choices per week	≤3 week choices per week
18	Folic acid supplementation	400 microgram per day folic acid supplementation 4 weeks before conception until 8 weeks after.	Supplements containing folic acid and/or folic acid from food		No supplementation/≥1,000 micrograms/day	400 up to 1,000 micrograms supplementation per day
19	Vitamin D supplementation	10 micrograms per day supplementation	Supplements containing vitamin D and/or vitamin D from food		No supplementation or ≥100 micrograms supplementation/day	10 up to 100 micrograms supplementation per day
20	Vitamin A	750 mcg RAE per day and not more than 3,000 mcg RAE per day.	Supplements containing vitamin A, liver products		0 or ≥3,000 mcg RAE per day	750 up to 3,000 mcg RAE per day
21	Iron	Consume sufficient products containing iron to obtain 12 mg per day	Iron from food and/or supplements		No iron intake	12 mg or more per day
#	Iodine	Consume sufficient products containing iodine to obtain 200 mcg per day	Bread, fish, dairy		No consumption of fish, dairy and bread	2 times a week fish, 3–4 portions dairy and 5 slices of bread per day

^*^Coffee and salt could not be included in DHD-P in this study resulting in 19 components included in this study (maximum score of 190). ^#^Iodine is not included in total score as it is calculated based on other components of the DHD-P namely fish, dairy and bread.

#### Dairy

Pregnant women are recommended to consume 3–4 portions of dairy products (13). Dairy and particularly calcium reduces the risk of pre-eclampsia, hypertension and pre-term birth (13, 22). Women score 10 points for a consumption between 3 and 4 portions daily. Aligning with the DHD-15 index, a maximum limit is set at 750 g/day, equivalent to approximately five portions per day according the Dutch portion size database portie-online.nl (23). Additionally, the Dutch Nutrition Centre recommends a maximum of 40 g of cheese per day (24). Pregnant women are recommended not to consume raw milk or cheese made from raw milk. Skimmed and semi-skimmed milk are part of the dietary recommendations of the Dutch Nutrition Centre, but full-fat milk is not. Butter is excluded from this category.

#### Fish

Pregnant women are recommended to consume fish twice weekly, including both lean and fatty fish, promoting brain development (25) and reducing the risk of early birth (13). The fish score is divided into two sub-scores; one for lean fish and one for fatty fish consumption. Earning 10 points requires a weekly 100-gram portion (approximately 15 g per day), with scores ranging from 0 to 10 for daily consumption between 0 and 15 g. Similarly, consuming at least 15 g per day of fatty fish is awarded 10 points, with a score ranging from 0 to 10 for daily consumption between 0 and 15 g. Both sub-scores equally contribute to the total fish score. Although it is crucial to avoid raw fish, particularly predator and pale fish, to minimize the risk of exposure to contaminants like dioxins and mercury (13), these specifics are not included in the score due to limited available information.

#### Caffeine

To promote fetal growth and prevent low birth weight (26), the Health Council recommends limiting caffeine intake to 200 mg per day (13), equivalent to two cups of coffee. Tea, with lower caffeine content, is allowed up to four cups (25). Exceeding these limits results in a score of 0, while intake below these thresholds earns a score of 10. The assessment uses cups of coffee as a proxy due to limitations in obtaining precise caffeine intake information through FFQ and 24-recall methods.

#### Alcohol

Alcohol poses risks to fertility and is strongly discouraged during pregnancy due to its association with birth defects and developmental disorders (27). The scoring system assigns 10 points to women who abstain from alcohol, while any amount of alcohol consumption results in a score of 0. The scoring reflects the unequivocal recommendation to completely avoid alcohol during pregnancy, as no safe threshold has been identified (13).

#### Soy products

The Nutrition Centre recommends women not to exceed four portions of soy drink or yoghurt per day and to limit soy product consumption to 2 times a week as part of dinner. No specific limits are set for women avoiding soy drink or yogurt. Soy contains isoflavones, which have a weak estrogenic effect, potentially having hormonal disruptive effects, and can pass the placenta (13). The upper intake limit is set at 1 mg per kg of body weight per day (13). Women surpassing the recommended limits receive 0 points, while those staying below are scored 10 points.

#### Folic acid supplementation

The Health Council of the Netherlands (HCN) recommends women actively planning, wishing, or experiencing unplanned pregnancies to use a daily folic acid supplementation of 400 mcg, starting 1 month before conception and continuing up to 10 weeks after conception (13). This is crucial as the recommended intake cannot be met through a regular diet, and supplementation reduces the risk on neural tube defects, early birth and low birth weight (13). Scoring ranges from 0–10: 10 points for intakes of 400 mcg or higher (up to 1,000 mcg based on Upper Tolerable Levels as set by the European Food Safety Authority) (28) in the first 10 weeks of pregnancy; 0 points for intakes of 0 or above 1,000 mcg; the score ranges between 0 and 10 for intakes between 0 and 400 micrograms and between 400 and 1,000 mcg the score ranges from 10 to 0. If a woman is more than 10 weeks pregnant, the score for this component is set at 10.

#### Vitamin D supplementation

Pregnant women, irrespective of skin color or sunlight exposure, are recommended to supplement their diet with 10 micrograms of vitamin D per day (29), which aligns with the general guidance for individuals with dark skin or low sun exposure. Vitamin D is important for bone growth of the baby, reduced risk of gestational diabetes and a low birth weight and asthma-like symptoms of the baby (13). Women score 10 points for an intake of 10 mcg vitamin D per day through supplements. A score of 0 is assigned for no intake or above 100 mcg (based on Upper Tolerable Levels as set by the European Food Safety Authority) (30), while scores between 0–10 and 100–10 mcg vary from 0 to 10.

#### Vitamin A

For pregnant women, the recommended daily intake of vitamin A is 750 mcg retinol-activity-equivalents (RAE), with a tolerable upper limit is 3,000 mcg per day (31). While vitamin A is essential for reproduction, excessive retinol intake can have teratogenic effects on the fetus. Women score 10 points for a 750 mcg RAE intake, and scores range from 0 to 10 for intakes between 0 and 750 mcg RAE and between 3,000 and 750 mcg RAE.

#### Iron

The Dutch Health Council recommends that pregnant women ensure sufficient dietary iron intake to prevent deficiencies (13). Sufficient iron intake levels reduce the risk of maternal anemia during and after pregnancy (32). Recognizing the challenge in obtaining adequate iron solely through diet, the recommended intake is set at 12 mg per day instead of the original 16 mg per day (13). Scoring awards 10 points for intakes of 12 mg or higher, with the score varying from 0 to 10 for intakes between 0 and 12 mg per day.

#### Iodine

The daily iodine requirement is 200 mcg per day (13), crucial for thyroid gland function, vital in the growth and brain development of the unborn child (13). To ensure adequate iodine intake, the Dutch Nutrition Centre recommends consuming fish twice a week, 3–4 portions of dairy per day, and 5 slices of bread daily (33). The iodine score, an additional score, is not included in the total score due to overlapping components. More specifically, the iodine score is calculated based on the subcomponents dairy, fish and bread, which each count for 1/3 of the score. The components dairy and fish are calculated as described earlier; consuming 5 slices of bread daily scores a 10, with the score increasing from 0 to 10 for 0 to 5 slices of bread daily.

#### DHD-P index in GLIMP-II

Diet quality scores based on FFQ and 24hR data were computed following the calculations outlined in Table 1. Due to limited dietary intake data obtained from both the FFQ and 24hR, some component scores were either not included in the final calculation or according to a modified approach based on available data. Specifically, coffee (lacking detail on being filtered/unfiltered) and salt intake (i.e., lacking detail on salt use in home cooked meals) scores could not be determined due to insufficient data. Excluding salt and coffee from the index resulted in a maximum possible score of 190 based on 19 components. Iodine was not included in the total score as it relies on other components in the index, namely dairy and fish.

#### Covariates

Body mass index (BMI) was calculated as body weight divided by squared body height (kg/m^2^). Body weight was measured to the nearest 0.1 kg with empty pockets and without shoes at each time point by trained professionals using a calibrated balance (SECA, Hamburg, Germany). Body height was measured to nearest 0.1 cm with no shoes on using a wall-mounted stadiometer (SECA, Hamburg, Germany). Standardized questions mainly based on the LifeLines study questionnaires (34) were employed to gather data on various maternal factors, including age (years), ethnicity (Western/non-Western), marital status (married/living together), parity (no/one or more children), educational level (low/mediate/high), smoking habits (yes/no), and physical activity (MET min/week) and history of gestational diabetes (yes/no). Ethnicity classification was determined based on the participant’s birth country and biological parents and was categorized in Western or non-Western. Education level was divided into low (primary school, vocational or lower general secondary education), mediate (higher secondary education or intermediate vocational training) and high (higher vocational education or university).

#### Statistical analysis

Categorical variables were depicted as frequencies and percentages. Median with interquartile range were shown for non-normally distributed data, while means with standard deviations were used for normally distributed data. Macronutrient levels were presented as percentage of total energy.

To assess relative validity, scores based on the FFQ were compared to scores based on the average of two 24hRs at 24 weeks of pregnancy by using a paired sample Wilcoxon Signed Rank Test for not normally distributed data. Spearman correlation coefficients were assessed using Lombards criteria (35) to indicate strength and direction of association of individual scores. A correlation coefficient below 0.20 was considered weak, between 0.20 and 0.49 moderate and 0.50 or higher good (35). Correlation coefficients between 24hR and FFQ intake levels were expected to be attenuated due to high within person variation from day-to-day variation of two 24hRs. To adjust for within person variation, de-attenuated correlation coefficients were calculated by using the method of Rosner and Willett (36).

To assess sensitivity to change, absolute scores were compared over time, and correlation coefficients were calculated between scores at 12 weeks and 24 weeks based on both FFQ and 24hR data (37). As data was not-normally distributed data, a paired Wilcoxon Signed Rank Test was performed and Spearman correlation coefficients were calculated.

Statistical analyses were performed using R 4.0.2 with a confidence level of 0.95.

## Results

Table 2 presents the baseline characteristics for the total population and stratified based on diet quality score. On average, women were 33 years old (SD: 3) with a BMI of 27 kg/m^2^ (SD: 5). Most women were married (84%), had children (96%), identified as western ethnicity (98%), and had a high education level (73%). Only 5% (*n* = 2) smoked, and 9% (*n* = 4) had a history of gestational diabetes. Although not statistically significant, there were noticeable differences in education between women with low and high DHD-P scores (59% versus 86% high educated, *p* = 0.11).

**Table 2:** Baseline characteristics of study population.

	Total	Low score^*^ (<113)	High score^*^ (≥113)	*p*-value^**^
Number of women [n]	47	24	23	
Age [mean (SD)]	33 (3)	32 (3)	33 (3)	0.29
BMI [mean (SD)]	27 (5)	27 (5)	27 (5)	0.90
Parity = 1 [*n* (%yes)]	42 (96)	21 (96)	21 (96)	1.00
Parity [median (Q1-Q3)]	1 (1–2)	1 (1–1)	1 (1–2)	0.58
Marital status [*n* (% married)]	37 (84)	17 (77)	20 (91)	0.41
Ethnicity [*n* (% western)]	43 (98)	22 (100)	21 (96)	1.00
Education				0.11
Primary [*n* (%)]	1 (2)	1 (5)	0 (0)	
Secondary [*n* (%)]	11 (25)	8 (36)	3 (14)	
Higher [*n* (%)]	32 (73)	13 (59)	19 (86)	
History of gestational diabetes mellitus [*n* (%yes)]	4 (9)	1 (5)	3 (14)	0.60
Smoking [*n* (%yes)]	2 (5)	2 (9)	0 (0)	0.47
Physical activity level (MET min/week)	1,185 (912)	1,239 (992)	1,127 (835)	0.68

^*^Low and high scores split on median scores based on FFQ data at 24 weeks gestation. ^**^p-value based on either chi-square test for categorical variables or *t*-test for continuous variables.

Percentages of total energy from macronutrients were comparable for FFQ and 24hR data at 24 weeks (Table 3). FFQ data indicated higher intakes of vegetables, legumes, total fish, lean fish, fatty fish, liquid fat and iron compared to the recall data, while the opposite trend was observed for vitamin A and caffeine intake. A significant difference was also observed when comparing FFQ and recall data for the total diet quality score, i.e., 113 (IQR: 100–126) and 104 (IQR: 96–109) (*p* < 0.001), respectively. Examining individual component scores showed that vegetables, fruit, legumes, fish, fats, caffeine, vitamin A and iodine scored significantly higher in FFQ compared to 24hR data. The processed meat score was higher in 24hR data compared to FFQ data [4 (IQR: 0–10) versus 3 (IQR: 0–5)]. All other components did not significantly differ between FFQ and 24hRs.

**Table 3:** Comparison of energy, macronutrients, and DHD-P components between 24hR and FFQ at 24 weeks gestation.

24 weeks	FFQ	24hR		FFQ	24hR			
	Absolute intake in grams/day			DHD-P score			FFQ vs. 24hR	
*n = 47*	Median (IQR)	Median (IQR)	*p*-value^#^	Score, median (IQR)	Score, median (IQR)	*p*-value^#^	Spearman rho (*p*-value)	De-attenuated Spearman rho^***^
Energy (kJ)	9,473 (7,951–10,892)	8,735 (7,807–10,272)	0.19					
Carbs (en%)	48 (46–50)	49 (45–51)	0.87					
Fat (en%)	34 (32–38)	36 (31–38)	0.89					
Protein (en%)	15 (14–16)	14 (13–16)	0.44					
Total diet score^*^				113 (100–126)	104 (96–109)	<0.001	0.55 (<0.001)	0.57
Vegetables	126 (86–215)	101 (42–177)	0.04	6.3 (4.3–10)	4.6 (2.1–8.2)	0.01	0.30 (0.04)	0.58
Fruit	218 (97–235)	190 (98–259)	0.30	10 (4.8–10)	9.5 (4.9–10)	0.04	0.72 (<0.001)	0.80
Grain products				6.0 (5.0–6.0)	5.0 (5.0–7.5)	0.46	0.30 (0.04)	0.34
Wholegrain products	131 (90–176)	105 (70–181)	0.34					
Refined grain products	66 (51–85)	64 (24–108)	0.88					
Legumes	7 (0–16)	0 (0–0)	<0.001	7.0 (0.0–10)	0.0 (0.0–0.0)	<0.001	0.44 (<0.01)	NA (<1)
Nuts	2 (0–6)	0 (0–0)	0.13	2.0 (0.0–4.0)	0.0 (0.0–0.0)	0.15	0.32 (0.03)	0.33
Dairy	317 (219–551)	330 (186–479)	0.23	6.3 (3.7–8.6)	7.3 (4.4–9.4)	0.32	0.24 (0.10)	0.25
Fish	27 (15–38)	0 (0–0)	<0.01	4.1 (2.3–5.7)	0.0 (0.0–0.0)	<0.001	0.09 (0.53)	0.18
Fatty fish	4 (0–9)	0 (0–0)	<0.001					
Lean fish	7 (5–13)	0 (0–0)	<0.001					
Liquid vs. solid fats ratio				10 (7.0–10)	0.0 (0.0–10)	<0.001	0.20 (0.19)	NA (>1)
Liquid fats	20 (8–29)	6 (0–18)	<0.001					
Solid fats	0 (0–2)	6 (2–18)	<0.001					
Red meat	40 (25–53)	15 (0–46)	<0.001	10 (8.5–10)	10 (10–10)	0.18	0.23 (0.12)	0.54
Processed meat	34 (23–48)	28 (0–62)	0.66	3.0 (0.0–5.0)	4.0(0.0–10)	0.05	0.42 (<0.01)	0.49
Sweetened beverages and fruit juices	185 (64–311)	167 (24–305)	0.62	3.0 (0.0–7.50)	3.0 (0.0–9.0)	0.46	0.57 (<0.001)	0.59
Alcoholic beverages	0 (0–0)	0 (0–0)	0.37	10 (10–10)	10 (10–10)	0.35	NA	NA
Caffeine (cups/day^**^)	2 (1–3)	2 (2–3)	<0.001	10 (0.0–10)	0.0 (0.0–10)	0.04	0.44 (<0.01)	0.47
Soy				10 (10–10)	10 (10–10)	NA	NA	NA
Soy drink	0 (0–0)	0 (0–0)	1					
Soy product	0 (0–0)	0 (0–0)	0.59					
Unhealthy choices (portions/day)	8 (5–11)	7 (5–10)	0.57	0.0 (0.0–4.60)	0.0 (0.0–5.4)	0.73	0.45 (<0.01)	0.48
Folic acid (mcg/day) supplements	400 (0–400)	NA	NA	10 (10–10)	NA	NA		
Vitamin D (mcg/day) supplements	5 (2–10)	NA	NA	5.0 (2.0–10)	NA	NA		
Vitamin A (mcg/day)	878 (704–1,098)	2,371 (1545–3,158)	<0.001	8.6 (7.5–9.4)	2.8 (0.0–6.2)	<0.001	0.37 (0.03)	0.40
Iron (mg/day)	23 (12–28)	22 (11–26)	<0.01	10 (9.8–10)	10 (8.8–10)	0.07	0.82 (<0.001)	0.91
Iodine^*^				5.1 (3.9, 6.5)	4.2 (3.1–5.2)	<0.001	0.41 (0.01)	0.45

^#^*p*-value from Wilcoxon Signed Rank Paired Test, ^*^total score is excluding iodine as iodine is calculated based on bread, fish and dairy intake and the coffee and salt components of the DHD-P could not be calculated, ^**^cups of coffee, with tea counted as ½ cup (as it contains half amount of caffeine compared to coffee), ^***^De-attenuated Spearman rho calculated following Rosner’s method. IQR, interquartile range; 95%CI, 95% confidence interval; kJ, kilojoule; RAE, retinol activity equivalent.

Despite significant absolute differences in the total diet quality score for the FFQ and recall, the de-attenuated correlation coefficient between the two methods was 0.57 and classified as good. Similarly, correlations for individual component scores when comparing FFQ and 24hR data were moderate or good for most components (Table 3). Specifically, correlations between component scores based on FFQ and 24hR data were moderate for whole grains, nuts, dairy, processed meat, caffeine, unhealthy choices, vitamin A and iodine (rho = 0.25–0.49) (35). For fruit, vegetables, sugar-sweetened beverages, red meat and iron correlations were good (>0.50). A low correlation was observed for fish (rho = 0.09). The uncorrected correlation coefficients for the total diet quality score, whole grain products, nuts, dairy, sweetened beverages and fruit juices, caffeine, unhealthy choices, vitamin A and iodine were similar to the de-attenuated values. No de-attenuated correlation coefficients could be calculated for legumes (Spearman rho = 0.44) and fat (Spearman rho = 0.20) due to high within person variation. A de-attenuated correlation coefficient for alcoholic beverages and soy products could not be calculated due the high similarity in consumption between FFQ and 24hR data, resulting in minimal variance and within person variation.

An overview of diet quality based on FFQ data at 12 and 24 gestational weeks is presented in Table 4, revealing significant absolute differences for the intakes of red meat [43 (IQR: 29–58) vs. 39 (IQR 23–47) g/day, *p* = 0.02], caffeine [2 (IQR: 1–2) versus 2 (IQR: 1–3) cups/day, *p* = 0.04] and supplemental folic acid [400 (IQR: 400–400) versus 400 (IQR: 0–400) mcg/day, *p* = 0.02]. No significant differences were observed for any other DHD-P components. Accordingly, evaluation of individual component scores did not significantly differ over time, except for red meat [diff = −0.9 (95%CI:−1.5; −0.3)], and the total diet quality score showed a correlation coefficient of 0.69 over time, with individual components correlations ranging from 0.27 for red meat to 0.84 for vegetables.

**Table 4:** Comparing median intake and DHD-P scores based on FFQ data at 12 and 24 weeks gestation.

FFQ	12 weeks	24 weeks		12 weeks	24 weeks			
Absolute intake in grams/day				DHD-P score				
*n = 27*	Median (IQR)	Median (IQR)	*p*-value^#^	Median (IQR)	Median (IQR)	Difference (95%CI)	*p*-value^#^	Spearman rho
Energy (kJ)	9,483 (8143–10,080)	9,606 (8375–10,790)	0.25					
Carbohydrates (e%)	48 (46–50)	48 (46–50)	0.35					
Fats (e%)	35 (33–36)	34 (32–37)	0.86					
Protein (e%)	15 (14–16)	15 (14–16)	0.16					
Total score^*^				134 (125–141)	133 (122–137)	1.2 (−3.6;5.9)	0.70	0.69 (<0.001)
Vegetables (g/day)	155 (102–241)	171 (106–238)	0.92	7.8 (5.1–10)	8.6 (5.4–10)	0.1 (−0.5;0.7)	0.79	0.84 (<0.01)
Fruit	222 (157–232)	224 (174–235)	0.13	10 (7.9–10)	10 (8.8–10)	0.2 (−0.8;0.4)	0.15	0.82 (<0.01)
Grain products				8.0 (6.0–10)	8.0 (6.0–10)	−0.1 (−1.1;0.9)	0.69	0.68 (<0.01)
Wholegrain products	140 (114–169)	133 (102–179)	0.82					
Refined grain products	83 (62–104)	77 (51–86)	0.19					
Legumes	11 (0–17)	7 (0–17)	0.99	10 (0.0–10)	7 (0–10)	0.4 (−1.2;2.0)	0.56	0.55 (<0.01)
Nuts	3 (0–7)	2 (0–5)	0.22	2.0 (0.0–5.0)	2.0 (0.0–3.5)	0.7 (−0.4;1.9)	0.27	0.61 (<0.01)
Dairy	341 (258–446)	374 (241–560)	0.06	7.6 (4.3–8.9)	6.3 (3.9–8.9)	0.8 (−0.8;2.3)	0.68	0.35 (0.07)
Cheese	29 (13–42)	30 (16–42)	0.82					
Fish	12 (10–17)	13 (8–21)	0.73	3.9 (3.5–5.6)	4.3 (2.8–6.7)	−0.2 (−1.3;0.8)	0.46	0.52 (0.01)
Fatty fish	4 (0–7)	4 (0–12)	0.92					
Lean fish	7 (5–12)	6 (5–12)	1.00					
Fats and oils				10 (10–10)	10 (10–10)	0.2 (−1.2;1.5)	0.89	0.53 (<0.01)
Liquid fats	24 (11–32)	21 (9–31)	0.59					
Solid fats	0 (0–0)	0 (0–1)	0.47					
Red meat	43 (29–58)	39 (23–47)	0.02	10 (8.0–10)	10 (10–10)	−0.9 (−1.5;-0.3)	<0.01	0.58 (<0.01)
Processed meat	43 (33–51)	38 (24–50)	0.50	1.0 (0.0–3.5)	2.0 (0.0–5.0)	−0.6 (−1.7;0.5)	0.49	0.46 (0.02)
Sweetened beverages and fruit juices	227 (55–362)	158 (53–272)	0.28	1.0 (0.0–8.0)	4.0 (0.0–8.0)	−0.7 (−2.2;0.8)	0.35	0.51 (<0.01)
Alcoholic beverages	0 (0–0)	0 (0–0)	NA	10 (10–10)	10 (10–10)	NA	NA	NA
Caffeine (cups/day^**^)	2 (1–2)	2 (1–3)	0.04	10 (0.0–10)	0.0 (0.0–10)	1.9 (−0.4;4.1)	0.11	0.37 (0.06)
Soy				10 (10–10)	10 (10–10)	NA	NA	NA
Soy milk/yoghurt	0 (0–0)	0 (0–0)	1.00					
Soy products	0 (0–0)	0 (0–0)	0.42					
Unhealthy choices	7 (5–10)	8 (5–10)	0.17	1.1 (0.0–4.9)	0.0 (0.0–4.3)	0.1 (−0.9;1.1)	0.84	0.69 (<0.01)
Folic acid from supplements	400 (400–400)	400 (0–400)	0.02	10 (10–10)	10 (10–10)	NA	NA	NA
Vitamin D (mcg/day) from supplements	5 (5–10)	5 (5–10)	0.85	5.0 (5.0–10)	5.0 (5.0–10)	0.6 (−1.5;1.7)	1.00	0.44 (0.02)
Vitamin A (RAE mcg/day)	967 (760–1,107)	878 (740–1,223)	0.80	8.6 (7.4–9.2)	8.6 (7.4–9.5)	−0.2 (−0.7;0.4)	0.59	0.31 (0.12)
Iron (mg/day)	24 (12–27)	24 (17–28)	0.95	10 (10–10)	10 (10–10)	−0.1 (−0.4;0.2)	0.86	0.20 (0.32)
Iodine*				5.4 (4.3–6.6)	5.3 (4.3–6.5)	0.3 (−0.3;0.9)	0.38	0.43 (<0.03)

^#^*p*-value from Wilcoxon Signed Rank Paired Test, ^*^total score is excluding iodine as iodine is calculated based on bread, fish and dairy intake and the coffee and salt components of the DHD-P could not be calculated, ^**^cups of coffee, with tea counted as ½ cup (as it contains half amount of caffeine compared to coffee). IQR, interquartile range; 95%CI, 95% confidence interval; kJ, kilojoule; RAE, retinol activity equivalent.

Consistent with the FFQ data, recall data showed a non-significant increase in energy intake over time (Table 5), with similar values for folic acid supplementation. In contrast to the FFQ data, recall data indicated lower refined grains [53 (IQR: 15–79) vs. 94 (IQR: 69–127)] and processed meat consumption at 24 weeks compared to 12 weeks gestation [24 (IQR: 0–49) and 41 (IQR: 14–70) g/d]. No significant differences in intakes were observed for any of the other DHD-P components. Accordingly, DHD-P scores did not substantially differ between 12 and 24 weeks, except for a higher score for grains at 24 weeks compared to 12 weeks [diff = 1.3 (95%CI: 1.0–2.5)]. The correlation coefficient for the total diet quality score between 12 and 24 weeks of pregnancy was 0.22 with correlation of individual components ranging between 0.02 for red meat and 0.69 for caffeine.

**Table 5:** Comparing median intake and DHD-P scores based on 24hR data at 12 and 24 weeks gestation.

Recalls	12 weeks	24 weeks		12 weeks	24 weeks			
Absolute intake in grams/day				DHD-P score				
*n = 27*	Median (IQR)	Median (IQR)	*p*-value^#^	Median (IQR)	Median (IQR)	Difference (95%CI)	*p*-value^#^	Spearman rho
Energy (kJ)	8,713 (7625–10,945)	8,934 (7890–10,396)	0.84					
Carbohydrates (e%)	50 (47–51)	50 (46–51)	0.54					
Fats (e%)	33 (32–35)	34 (31–37)	0.81					
Protein (e%)	14 (13–16)	14 (13–16)	0.23					
Total score^*^				101 (93–118)	105 (98–109)	1.3 (−5.6;8.2)	0.53	0.22 (0.28)
Vegetables (g/day)	122 (49–167)	103 (61–163)	0.97	6.1 (2.5–8.4)	5.1 (3.0–8.2)	0.2 (−1.2;1.6)	0.89	0.46 (0.02)
Fruit	167 (92–266)	205 (98–290)	0.61	8.4 (4.6–10)	10 (4.9–10)	0.4 (−1.1;1.9)	0.54	0.46 (0.02)
Grain products				5.0 (4.0–6.0)	5.0 (5.0–10)	1.3 (1.0;2.5)	0.03	0.42 (0.03)
Wholegrain products	123 (70–154)	123 (88–181)	0.36					
Refined grain products	94 (69–127)	53 (15–79)	0.05					
Legumes	0 (0–0)	0 (0–0)	0.29	0 (0–0)	0 (0–0)	−1.1 (−2.7;0.5)	0.23	0.10 (0.64)
Nuts	0 (0–0)	0 (0–0)	0.79	0 (0–0)	0 (0–0)	−0.3 (−1.6;0.9)	0.68	0.45 (0.02)
Dairy	196 (80–332)	203 (97–313)	0.82	6.8 (4.7–9.7)	7.3 (4.7–9.6)	0.1 (−1.2;1.4)	0.68	0.39 (0.04)
Cheese	30 (11–44)	30 (20–35)	0.76					
Fish	0 (0–0)	0 (0–0)	0.56	0.0 (0.0–0.0)	0.0 (0.0–1.0)	0.1 (−1.0;1.2)	1.00	0.10 (0.60)
Fatty fish	0 (0–0)	0 (0–0)	0.59					
Lean fish	0 (0–0)	0 (0–0)	0.40					
Fats and oils				2.0 (0.0–10)	1.0 (0.0–8.5)	−0.7 (−2.6;1.2)	0.43	0.51 (0.01)
Liquid fats	15 (2–21)	7 (3–20)	0.46					
Solid fats	5 (0–16)	6 (1–18)	0.88					
Red meat	0 (0–29)	0 (0–45)	0.63	10 (10–10)	10 (10–10)	0.1 (−0.9;1.2)	0.92	0.02 (0.92)
Processed meat	41 (14–70)	24 (0–49)	0.02	2.0 (0.0–7.5)	5.0 (0.5–10)	1.6 (−0.3;3.6)	0.11	0.30 (0.13)
Sweetened beverages and fruit juices	200 (0–355)	150 (13–305)	0.93	2.0 (0.0–10)	4.0 (0.0–9.5)	0.1 (−1.9;2.2)	0.97	0.25 (0.21)
Alcoholic beverages	0 (0–0)	0 (0–0)	1	10 (10–10)	10 (10–10)	−0.4 (−1,1;0.4)	1	NA
Caffeine (cups/day^**^)	3 (2–4)	3 (2–3)	0.56	0.0 (0.0–10)	0.0 (0.0–0)	−0.7 (−2.3;0.8)	0.42	0.63 (<0.01)
Soy				10 (10–10)	10 (10–10)	0	NA	NA
Soy milk/yoghurt	0 (0–0)	0 (0–0)	NA					
Soy products	0 (0–0)	0 (0–0)	1					
Unhealthy choices	7 (4–8)	7 (5–10)	0.68	0.5 (0.0–7.5)	0.0 (0.0–5.6)	−0.3 (−1.9;1.3)	0.93	0.56 (<0.01)
Folic acid from supplements	400 (400–400)	400 (0–400)	0.02	10 (10–10)	10 (10–10)	NA	NA	NA
Vitamin D from supplements	5 (5–10)	5 (5–10)	0.85	5 (5–10)	5 (5–10)	−0.1 (−1.7;1.5)	1	0.44 (0.02)
Vitamin A (mcg/day)	2,762 (2456–3,751)	2,702 (1990–3,317)	0.16	1.1 (0.0–2.4)	1.3 (0.0–4.5)	0.7 (2.2;-0.8)	0.39	0.12 (0.55)
Iron (mg/day)	22 (12–27)	23 (14–26)	0.43	10 (9.9–10)	10 (10–10)	0.2 (−0.3;0.8)	0.41	0.21 (0.30)
Iodine^*^				4.0 (3.0–4.3)	4.3 (3.5–5.6)	0.4 (−0.4;1.2)	0.34	0.02 (0.92)

^#^*p*-value from Wilcoxon Signed Rank Paired Test, ^*^total score is excluding iodine as iodine is calculated based on bread, fish and dairy intake and the coffee and salt components of the DHD-P could not be calculated, ^**^cups of coffee, with tea counted as ½ cup (as it contains half amount of caffeine compared to coffee). IQR, interquartile range; 95%CI, 95% confidence interval; kJ, kilojoule; RAE, retinol activity equivalent.

## Discussion

In this study, we developed the DHD-P index using the recommendations from the Dutch Health Council for pregnant women and evaluated it using GLIMP-II study data. Despite significant differences for several absolute intake estimates and individual component scores when comparing FFQ and 24hR data at 24 weeks gestation, de-attenuated correlation coefficients were moderate or good for almost all components. Moreover, FFQ and recall data both showed comparable dietary intake data for daily consumed food groups at 12 and 24 weeks, suggesting minimal changes during pregnancy. Further, this study demonstrated moderate or good correlations over time of the adjusted DHD score based on FFQ data compared to 24hRs data among Dutch pregnant women indicating good reproducibility.

Various diet quality indexes for pregnant women have been developed worldwide, using different dietary guidelines and scoring methods. For instance, the Diet Quality Index for Pregnancy (DQIP) including eight components was developed by Bodnar and Siega-Riz (10) based on FFQ data of 2,063 women in the second trimester while applying the US dietary guidelines. In contrast to the DHD-P’s different scoring for folic acid supplementation based on the specific stage of pregnancy, the DQIP maintained consistent scoring across trimesters. In terms of the number of food groups assessed in previously developed indices, our index markedly differs from a Brazilian diet quality score, developed by Crivellenti et al. (11). Whereas our index consists of 14 food groups and eight nutrient components, the Brazilian index is composed of five nutrient components, three food group components and one moderator component reflecting the percentage of total energy from ultra-processed foods (11). Kennedy et al. (8) developed an index based on EFSA, WHO and USA Institute of Medicine guidelines, which scored the adherence to the guidelines for each nutrient or food group with 1 point, weighting all food groups and nutrients the same. Further, the Healthy Food Intake Index containing 11 components based on the Nordic Nutrition Recommendations was scored ranging between 0 and 1 or 0 and 2 depending on the relevance for overall diet quality (9). Conversely, the DHD-P applies an equal weighting approach for all its components due to the challenges in assigning weights to components based on health effects. Variations in indices can be explained by variations in the underpinning recommendations, divergence in the employed dietary assessment methodologies during index development, and the prioritization choices made by developers (e.g., specific emphasis on certain food groups, nutrients, or health outcomes).

In this study, we observed median diet quality scores of 133 and 134 out of 190 based on FFQ data at 12 and 24 weeks gestation. In comparison, Nguyen et al. (38) observed a mean diet quality score of 7.6 out of 15 among 4,824 Dutch pregnant women who completed an FFQ between 2002 and 2006. They used a scoring system based on the 2015 Dutch dietary guidelines for adults, incorporating folic acid supplements, and assigning a maximum score for alcohol abstinence (38). Thus, scores in our study were higher than the scores observed by Nguyen et al. (38), which may potentially be explained by a higher percentage of high educated women in the GLIMP-II study (73% versus 47%). Further, we observed a good correlation between the diet quality index calculated with the FFQ and 24hRs, which is highly comparable to the results of the evaluation of the original DHD-index among 885 adults (5). Most component specific coefficients between the two methods were also comparable between our study and the original DHD-evaluation, including lower correlation coefficients for episodically consumed foods. Considering the absolute intake levels, the FFQ data of our study showed higher intake levels for 7 out of 28 nutrients or food groups compared to the average of the two 24hRs, particularly for some episodically consumed foods such as fish and legumes. A meta-analysis including 130 studies evaluating validity of the FFQ for epidemiological studies among adults (39), also showed higher intake levels for 17 out of 32 nutrients for FFQ data vs. recall data. Moreover, a validation study on the FFQ used in this study observed higher reported levels of 8 out of 30 nutrients compared to recall data (17). In light of the inherent attributes of the two methodologies, the observed outcome in our study is not unexpected. The FFQ is anticipated to more effectively capture habitual food intake when contrasted with two 24-h dietary recalls. As a result, the FFQ is expected to provide a more accurate estimation of the habitual intake pertaining to episodically consumed foods (5). Given that the total diet quality score and most individual component scores exhibited moderate or good correlation when comparing FFQ and 24hR data, we consider the DHD-P index a useful tool to compare scores between pregnant women and for self-monitoring (35).

When comparing diet quality between 12 and 24 weeks gestation, we observed limited variation in absolute intake levels and diet quality component scores of commonly consumed food groups for both FFQ and 24hR data indicating minimal dietary changes during pregnancy. Particularly if differences existed these are expected to become evident using 24hR data as these provide more detailed dietary information compared to FFQs. It can be questioned whether diet remains completely similar during pregnancy trimesters as, e.g., women may be more motivated to eat healthy at the beginning of pregnancy (40, 41), but many other factors, such as physical complaints, could play a role in dietary habits during pregnancy as well (41). Although only a limited number of studies explored dietary intake at different stages during pregnancy, a study among about 2,500 UK pregnant women also observed minimal alterations in dietary patterns (42). In line, no changes in overall diet quality were observed over pregnancy trimesters among 79 Canadian pregnant women. However, in terms of individual components, intakes of fruit and vegetables decreased and ‘milk and alternatives’ increased during pregnancy among the Canadian women (41). Based on the FFQ data of our study, we observed a similar trend for dairy intake. In terms of reproducibility, FFQ data showed moderate to good correlations, while those based on 24hRs were categorized as low to moderate according to Lombard’s criteria (35, 43). It is essential to emphasize that correlation coefficients signify the relatedness between measurements over time, not the level of agreement (44). Consequently, this suggests that scores over time based on FFQ are more closely intertwined compared to those based on 24hR data. This difference may again be attributed to substantial day-to-day variation, particularly for episodically consumed foods like fish, legumes, and associated nutrients (e.g., iodine) when relying on only two 24hRs.

So far, a few diet quality scores exist for Dutch pregnant women, but these were not evaluated and are not based on the recent guidelines. We used data from the GLIMP-II study including intake levels based on both FFQ and two 24hRs at two time points during pregnancy to exploratively evaluate the DHD-P index. While correlation coefficients can assess reproducibility and indicate the validity of long-term dietary intake measures, it is crucial to recognize that high reproducibility does not necessarily ensure high validity (43). Limitations of this study are its limited sample size, and the inclusion of women with a predisposition for gestational diabetes, reducing external validity to the generally pregnant population in the Netherlands. Additionally, both 24hRs and FFQ have its limitations in assessing habitual dietary intake as they both rely on memory and a food composition table to calculate nutrient values. Furthermore, the 24hRs used in GLIMP-II were web-based, which likely resulted in lower reporting rates as no additional questions could be asked in comparison to when performed during a phone call or meeting in person. Additionally, the FFQ used in this study was not designed to inquire about nutrients or food groups specifically of interest during pregnancy, such as vitamin A consumption via liver products. Distinguishing between types of products (e.g., low or high fat milk) in FFQs depends on question specificity. Despite attempts to score similar food products in both 24hR and FFQ data, differences may have occurred from the FFQ’s broader product inquiries compared to the 24hRs. Another limitation of the current evaluation study is the inability to calculate the intake of all intended components, such as filtered coffee and salt intake. To note, the Eetscore tool, developed by researchers from Wageningen University, can be used as a diet screener to collect all this necessary dietary data to calculate DHD-15 scores (45). Therefore, using the updated Eetscore tool in combination with the DHD-P potentially provides the optimal assessment method for diet quality among Dutch pregnant women.

## Conclusion

This study presents the development of the DHD-P index based on Dutch dietary guidelines for pregnant women issued by the Dutch Health Council in 2021. Additionally, we performed an explorative evaluation on the DHD-P to provide insight in the index’s usability and reproducibility. The DHD-P appears to be an appropriate index to assess dietary quality among pregnant women, and could serve as a foundation to provide dietary feedback toward healthier food choices. Further evaluations using more complete dietary data specifically relevant in respect to the dietary guidelines for pregnant women and the DHD-P, such as salt intake and type of coffee consumption, are recommended.

## Data availability statement

The data analyzed in this study is subject to the following licenses/restrictions: the raw data supporting the conclusions of this article will be made available by the authors, upon reasonable request. Requests to access these datasets should be directed to elske.brouwer-brolsma@wur.nl.

## Ethics statement

The studies involving humans were approved by Medical Ethics Committee of Wageningen University & Research. The studies were conducted in accordance with the local legislation and institutional requirements. The participants provided their written informed consent to participate in this study.

## Author contributions

JF: Conceptualization, Formal analysis, Investigation, Methodology, Visualization, Writing – original draft, Writing – review & editing. EF: Data curation, Funding acquisition, Resources, Supervision, Writing – review & editing. EB-B: Conceptualization, Data curation, Formal analysis, Funding acquisition, Investigation, Methodology, Resources, Supervision, Writing – original draft, Writing – review & editing.
